# Measuring thermal spread during bipolar cauterizing using an experimental pneumoperitoneum and thermal sensors

**DOI:** 10.3389/fsurg.2023.1115570

**Published:** 2023-06-13

**Authors:** Salvatore Siracusano, Giacomo Marchioro, Dumitru Scutelnic, Matteo Brunelli, Renato Talamini, Antonio Benito Porcaro, Paolo Fiorini, Riccardo Muradore, Claudia Daffara

**Affiliations:** ^1^ Department of Life, Health and Environmental Sciences, University of L’Aquila, L’Aquila, Italy; ^2^ Department of Computer Science, University of Verona, Verona, Italy; ^3^ Department of Diagnostics and Public Health, University of Verona, Verona, Italy; ^4^ Consultant Epidemiologist CRO Aviano, Aviano, Italy; ^5^ Department of Urology, University of Verona, Verona, Italy

**Keywords:** experimental pneumoperitoneum, bipolar cauterizing, thermal energy spread, infrared endoscopic camera, sealed plexiglass chamber

## Abstract

**Objective:**

During nerve-sparing robot-assisted radical prostatectomy (RARP) bipolar electrocoagulation is often used but its use is controversial for the possible thermal damage of neurovascular bundles. Aim of the study was to evaluate the spatial-temporal thermal distribution in the tissue and the correlation with the electrosurgery-induced tissue damage in a controlled, CO2-rich environment modelling the laparoscopy conditions..

**Methods:**

We manufactured a sealed plexiglass chamber (SPC) equipped with sensors to reproduce experimentally the environmental conditions of pneumoperitoneum during RARP. We evaluated in 64 pig musculofascial tissues (PMTs) of approximately 3 cm^3^ × 3 cm^3^ × 2 cm^3^ the spatial-temporal thermal distribution in the tissue and the correlation with the electrosurgery-induced tissue damage in a controlled CO2-rich environment modeling the laparoscopy conditions. Critical heat spread of bipolar cauterizing during surgical procedure was assessed by the employment of a compact thermal camera (C2) with a small core sensor (60 × 80 microbolometer array in the range 7–14 μm).

**Results:**

Bipolar instruments used at 30 W showed a thermal spread area of 18 mm^2^ when applied for 2 s and 28 mm^2^ when applied for 4 s. At 60 W, bipolar instruments showed a mean thermal spread and 19 mm^2^ when applied for 2 s; and 21 mm^2^ when applied for 4 s. Finally, histopathological analysis showed that thermal damage is distributed predominantly on the surface rather than in depth.

**Conclusions:**

The application of these results is very interesting for the definition of an accurate use of bipolar cautery during nerve-sparing RARP. It demonstrates the feasibility of using miniaturized thermal sensors, thus addressing the potential for next developments regarding the design of thermal endoscopic devices for robotic use.

## Introduction

It is well known that robot-assisted laparoscopic (RAL) procedures now represent the gold standard for most urological procedures since the great advantage of robotic surgery is the three-dimensional vision that requires continuous hemostasis to maximize the clear vision of the operating field. In this way, the objective of nerve-sparing robot-assisted radical prostatectomy (RARP) is the removal of the prostate without damage or transecting the neurovascular bundles (NVBs) that are intimately localized to the lateral, posterolateral, and posterior surface of the prostate (1). At present, bipolar electrocoagulation or ultrasonic energy is often used to obtain the hemostasis, which occurs when the temperature rises above 45°C (2, 3); however, denaturation of proteins with cell death is achieved for temperatures between 57°C and 65°C (4).

The evaluation of thermal energy spread has already been addressed in the past in the literature but exclusively in experimental conditions of open surgery (5). In this regard, Goldstein (6) analyzed the tissue response to surgical energy with special reference to Harmonic ACE, bipolar Gyrus Trisector, and LigaSure V showing that the damage of the adjacent tissue is not only related to the temperature of the blade but mainly to the energy application time. In particular, they demonstrated that LigaSure V was superior in sealing large vessels but at large energy spread compared with the Harmonic ACE. At the same time, Brzeziński et al. (7) analyzed the lateral thermal spread during thyroidectomy when using monopolar or bipolar diathermy by comparing 30 and 50 W settings. In this case, the infrared camera imaging showed a higher heat spread for all instruments in the 60 W setting compared with the 30 W setting. Subsequently, Hefermehl et al. (8) showed that bipolar devices show significant spreads around 3 mm and, at the same time, that the positioning of Maryland forceps next to the heat-generating devices reduces the heat spread. Recently, Neckay et al. (9) showed that the application time is critical in thermal spread during appendectomy aided by monopolar energy because tubular anatomic structures can enhance thermal injury on distant tissues.

In this context, the use of bipolar electrocoagulation during RARP is still controversial because it is often associated with high rates of erectile dysfunction, urinary incontinence, and worsening of quality of life (10, 11). In the past, the critical thermal spread coagulation of devices employed in robotic surgery was tested but without observing its behavior in the course of pneumoperitoneum involving the use of carbon dioxide (8, 12, 13).

In the present study, we manufactured a sealed plexiglass chamber equipped with sensors for reproducing experimentally the environmental conditions of a pneumoperitoneum employed during RARP in order to assess the critical heat spread of bipolar cauterizing used during this surgical procedure by the employment of a compact thermal camera. Miniaturized thermal sensors available nowadays can be integrated in novel devices (i.e., endoscopic) suitable to be used in a real (i.e., laparoscopic) environment (14).

The aim of this study was to evaluate the spatial-temporal thermal distribution in the tissue and the correlation with the electrosurgery-induced tissue damage in a controlled, CO_2_-rich environment modeling the laparoscopy conditions.

## Materials and methods

### Experimental pneumoperitoneum by laparoscopic simulator chamber and monitoring sensors

**Figure 1:**
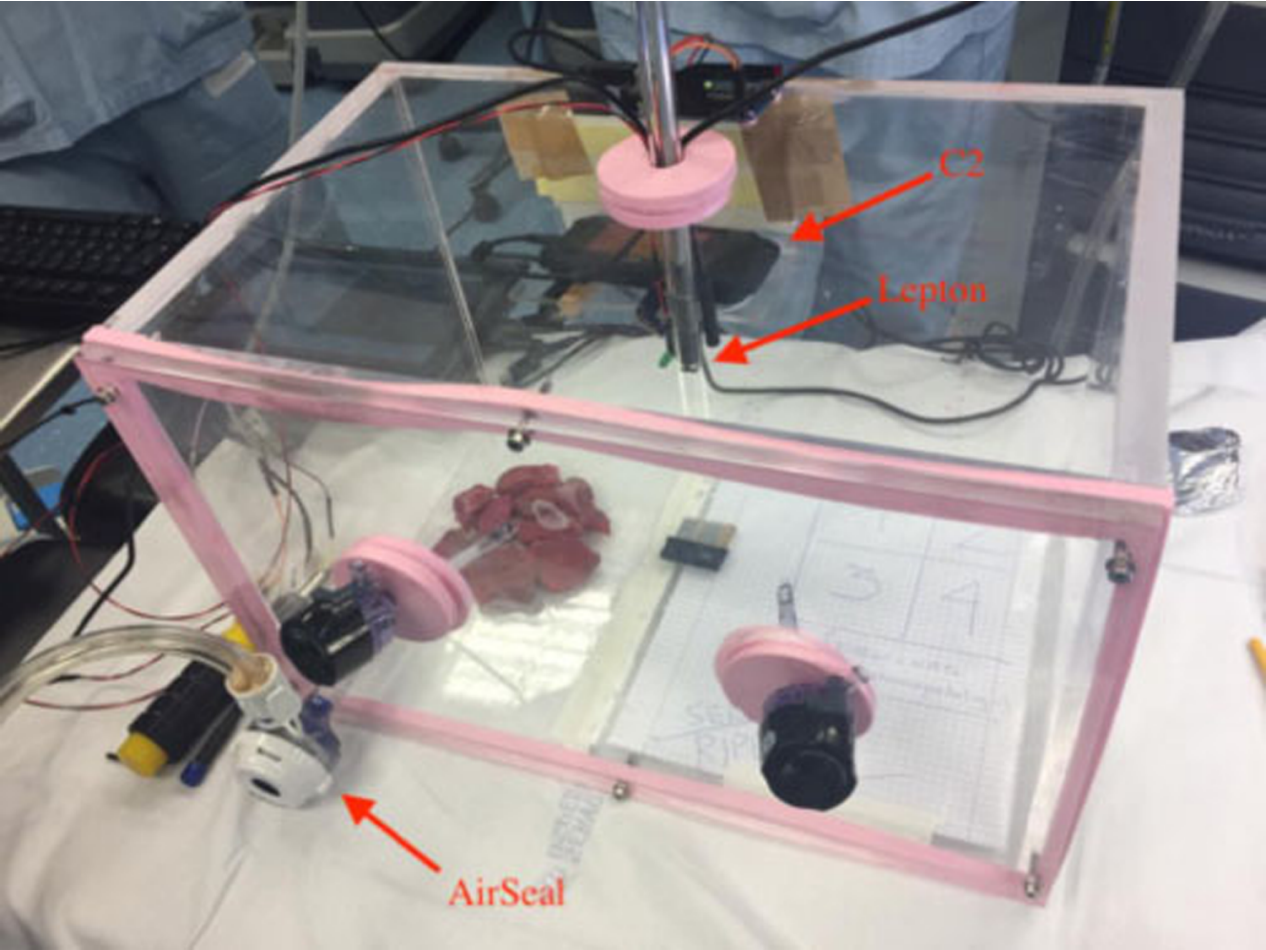
The SPC reproducing environmental conditions of pneumoperitoneum. The AirSeal and the C2 thermal camera are highlighted together with an additional thermal sensor (Lepton by FLIR) inserted in a trocar.

A sealed plexiglass chamber (SPC) was manufactured to reproduce the environmental conditions of the pneumoperitoneum during RAL, in constant pressure and temperature control of CO_2_ at 12 mmHg and at 37°C (Figure 1). The SPC has a dimension of 25 cm × 25 cm × 40 cm and was built using six plexiglass slabs with thickness ranging from 10 to 15 mm produced using an industrial laser cutter. Five of them were sealed using 1,2-Dichloroethane while one side was removable allowing the insertion of the specimens and the instruments and could be closed using six screws. We used custom silicone gaskets for allowing the insertion of the trocars and mimicking the behavior of human tissue.

The SPC is equipped with monitoring sensors. We placed an environment sensor (BMP280 Digital Pressure Sensor manufactured by Bosch) that provided pressure measurements with an accuracy of ±1 hPa and temperature reading with an accuracy of ±0.5°C. The pneumoperitoneum pressure was kept constant by the CO_2_ insufflator.

Four trocars were positioned inside the SPC, two were used for the insertion of the Maryland forceps (one of them electrified in bipolar mode), and the third one for the positioning of the air-real system. The additional fourth trocar of the chamber was designed to host an endoscopic vision system (14).

A FLIR™ C2 thermal camera (C2) was used for recording the temperature of the samples during the experiments. The C2 is a compact thermal camera with an array of 60 × 80 microbolometers sensitive in the range of 7–14 μm with a thermal accuracy of ±2°C and thermal sensitivity <0.10°C. The camera was placed in a fixed position inside the SPC and connected to an external laptop for recording in continuous the sequence of thermograms; the research-grade software FLIR™ ResearchIR was used to control the thermal acquisition.

**Figure 2:**
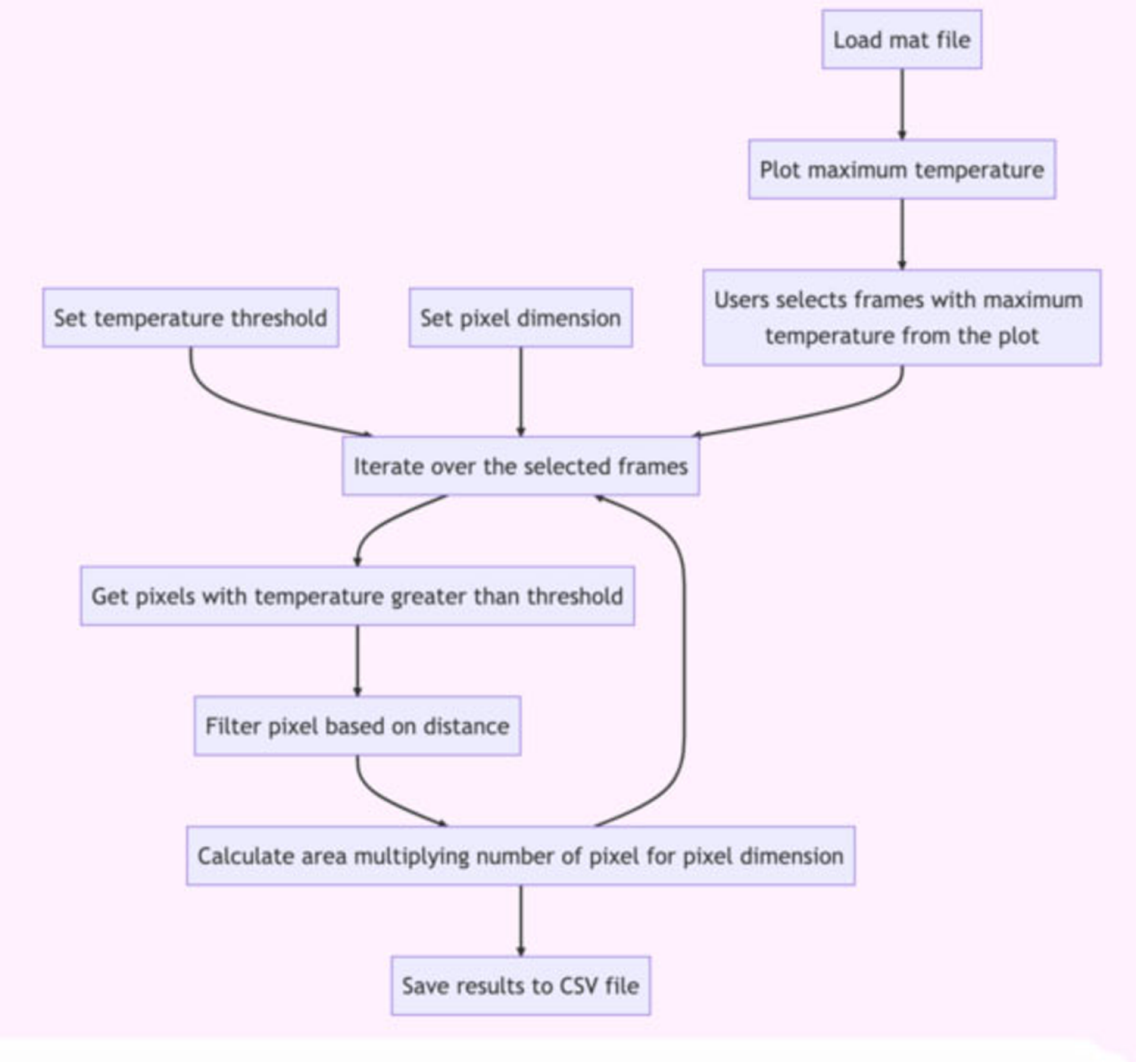
Diagram of the algorithm for the computation of the thermal spread from the thermal sequence.

The diagram in Figure 2 depicts the algorithm for handling the thermal data and computing the thermal spread.

### Experiment design

**Figure 3:**
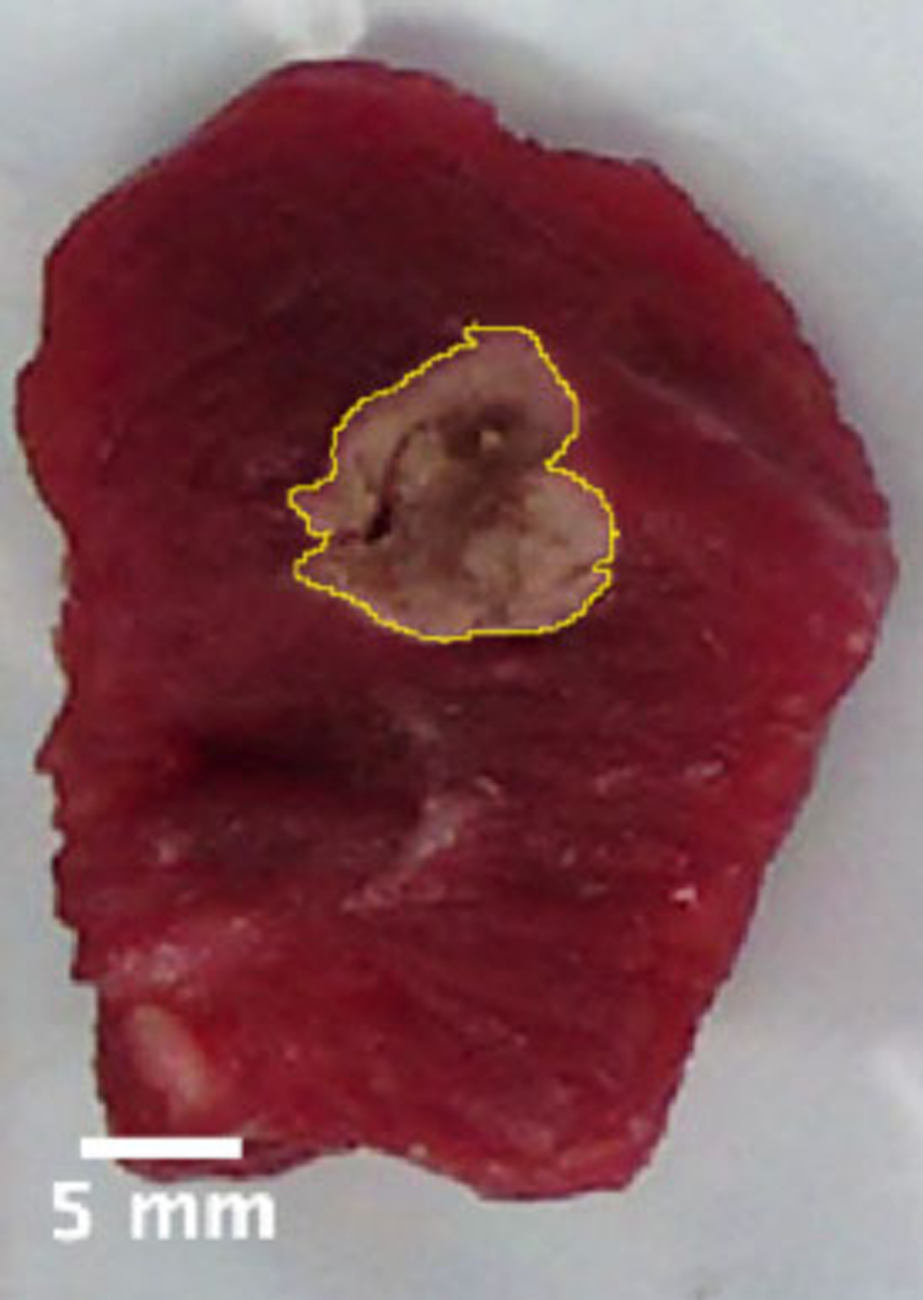
Example of delimitation of the tissue damage by macrophotography.

Bipolar electrocautery was performed using an Olympus ESG-400 electrosurgical generator set in power coag. effect 3. We used the electrocautery on 64 pig musculofascial tissues (PMTs) of approximately 3 × 3 × 2 cm^3^ and for which no animal sacrifice was necessary. We tested different application times (second) of the scalpel and power settings (Watt) of the electrosurgical generator: 2 s at 30 W, 2 s at 60 W, 4 s at 30 W, and 4 s at 60 W. The area (mm^2^) of thermal spread were calculated and compared to the macroscopic surface area of tissue damage (Figure 3). We combined the imaging data from the thermal camera, macrophotography, and microscopy for calculating the surface area and the depth of the tissue affected by the thermal stress. The details of the calculation are reported in the following sections.

We rebuilt the 3D tissue sections by using the Grundium Ocus digital microscope and compared the visual hematoxylin and eosin (H&E) evaluation with to the virtual ex-glass slides proposed by the digital instrument.

### Calculation of damaged area due to lateral thermal spread

The damaged area induced by the thermal stress was evaluated from macrophotography using a commercial camera and compared with the data provided by the C2 thermal camera. In the case of macrophotography, we selected the tissue area affected by the thermal stress based on the visual inspection of the images. Using ImageJ software (15), for each sample, we delimited the region affected by the necrosis as shown in Figure 3.

**Figure 4:**
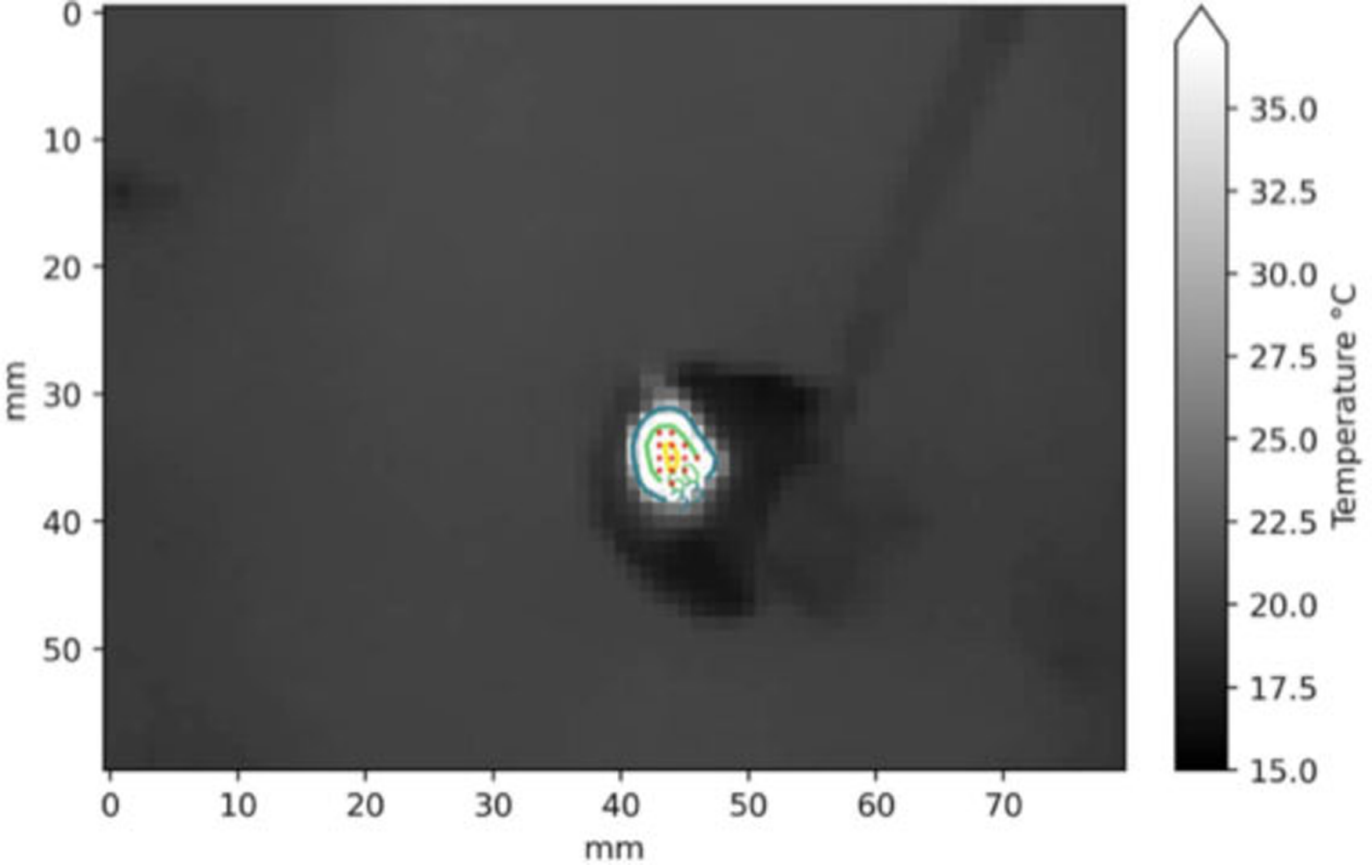
Example of segmentation in a frame of the thermogram sequence (2 s at 30 W setting). The red dots are the pixels with temperature higher than 60°C.

For computing the damaged tissue area from the sequence of thermograms produced by the C2, we created an algorithm for selecting the pixels of the thermograms that reported a temperature greater than 60°C (Figure 4). We used a metric reference placed inside the SPC with a known dimension for calculating the effective size of the sampled pixel in the tissue.

### Determination of the tissue damaged depth by means of histopathological analysis

Immediately after electrosurgery, the sample materials were formalin-fixed and paraffin-embedded. H&E staining was utilized and 5-μm serial section has been provided on tissue glass slides for further evaluation. We interpreted positive tissue damage, as ischemic and necrotic area with loss of vital tissue characters, according to details visible at optical microscope as follows: loss of anatomic architecture, loss of organ profile, proteinaceous degeneration, presence of amorphous cytoplasm, loss of nuclear details, and loss of cellular profile. The evaluation was performed by an expert surgical pathologist.

### Statistical analysis of the data

We used the Wilcoxon rank test for assessing the distributions of the data collected for the combination of the setting parameters time (second) and power (Watt).

For assessing the correlation between the damaged areas recorded with the different techniques, we used the Spearman's rank correlation coefficient. Spearman analysis includes monotonic relationships between variables.

## Results

The experiment was performed on a total of 64 PTM samples prepared in similar volumes of approximately 3 cm × 3 cm × 2 cm, divided in four separate groups of 16 measurement sessions for the different settings. The combination of power of 30 and 60 W application time of 2 and 4 s was considered.

All the sessions were carried out using the experimental pneumoperitoneum in an environment with controlled CO_2_ pressure reproducing the conditions of the surgical operation.

**Figure 5:**
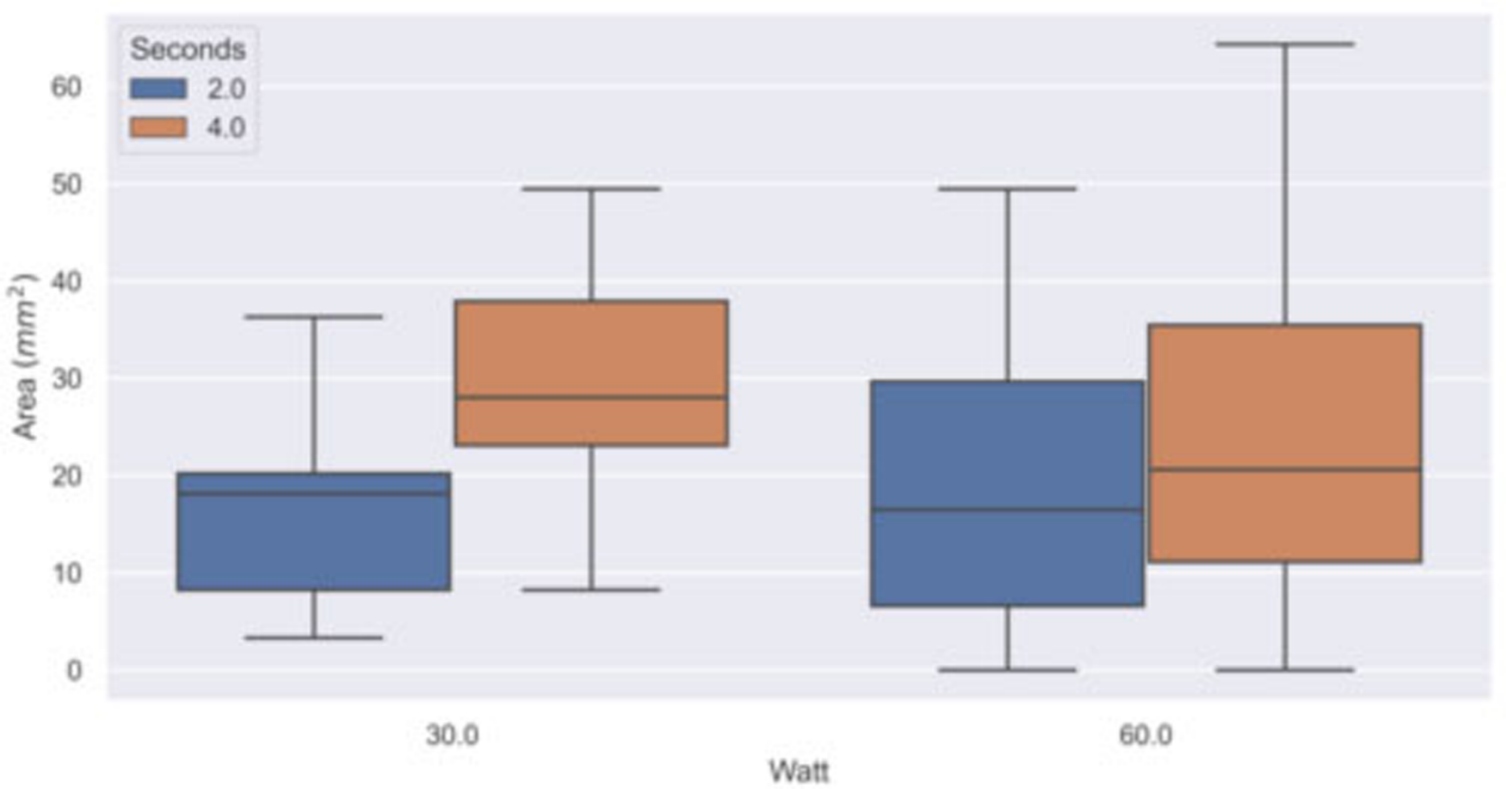
Boxplot of the experimental dataset. The area with temperature higher than 60°C (thermal spread) was computed in groups of samples by power (Watt) and time (second) settings using the thermal sequences acquired with the C2 thermal camera.

In Table 1, the results obtained with the C2 thermal camera are shown, while Figure 5 reports the boxplot analysis of the experimental datasets.

**Table 1:** Median and interquartile range (IQR) of the areas with temperature higher than 60°C (thermal spread) computed in groups of samples (*N*) by power (Watt) and time (second) settings, using the C2 thermal camera.

Variables	Time: 2 s		Time: 4 s	
	*N*	Median (IQR)	*N*	Median (IQR)
Thermal spread (mm^2^)				
Power: 30 W	16	18 (12)	15	28 (15)
Power: 60 W	14	19 (23)	15	21 (24)
*p-value* ^a^	* *	*0.3815*	* *	*0.7775*

^a^ Wilcoxon rank test, *p*-value between groups 30 and 60 W.

The thermal spread for each setting was estimated as the median of the areas computed in the single measurement sessions, by performing a pixel segmentation with a threshold of 60°C in the entire thermal sequence. The scalpel tip was used as a metric reference, thus allowing us to obtain the reliable pixel size for each measurement session. The interquartile range (IQR = *Q*_3_ − *Q*_1_) of the distributions and the number of trials (*N*) are indicated. Some measurements were rejected due to problems occurred in the data synchronization/recording workflow.

Bipolar instruments used at a power of 30 W showed a thermal spread area distribution with a median of 18 mm^2^ (IQR: 12 mm^2^) when applied for 2 s and of 28 mm^2^ (IQR: 15 mm^2^) when applied for 4 s. At 60 W, bipolar instruments showed a thermal spread distribution with a median of 19 mm^2^ (IQR: 23 mm^2^) when applied for 2 s and of 21 mm^2^ (24 mm^2^) when applied for 4 s. The pixel size of the thermal image at the tissue plane was about 1 mm^2^. The observed IQR values indicate a large variation in the samples, especially for the 60 W power setting. The thermal diffusion is more rapid at 60 W than at 30 W because of the different initial temperature gradient between the scalpel and the samples.

The Wilcoxon rank test between the statistical distributions shows no significant difference between the power settings 30 and 60 W at time applications 2 and 4 s (*p* = 0.3815 and *p* = 0.7775, respectively). If we perform the Wilcoxon test on each subgroup, we observe a *p*-value of 0.004 and 0.35 between the distribution at 2 and 4 s at 30 and 60 W, respectively, and *p*-values of 0.49 and 0.63 between the distribution at 30 and 60 W obtain at 2 and 4 s, respectively. The tests are consistent with the results shown in Table 1.

Table 2 shows the results of the histopathological analysis. The depth of thermal damage in tissue is estimated as mean for each setting. Bipolar instruments at 30 W showed a mean depth of 1.1432 mm when applied for 2 s and of 1.0012 mm when applied for 4 s. At 60 W, bipolar instruments showed a mean depth of 1.3221 mm when applied for 2 s and of 1.4117 mm when applied for 4 s. The spread of the damage on tissue (depth of damage) ranged from 0.6 to 1.7 mm. The damage was observed on all tissue sections, serially built using steps of 5 to 5 μm one to one.

**Table 2:** Histopathological analysis: mean and standard deviation (±SD) of depth of thermal damage (mm) in groups of samples (*N*.) by power (watt) and time (second) settings.

Variables	Time: 2 s		Time: 4 s	
	*N*	Mean (±SD)	*N*	Mean (±SD)
Depth of damage (mm)				
Power: 30 W	15	1.1432 (±0.0112)	14	1.3221 (±0.0324)
Power: 60 W	13	1.0012 (±0.0109)	16	1.4117 (±0.0127)
*p-value* ^a^	* *	*0.4149*	* *	*0.8345*

^a^ Wilcoxon rank test, *p*-value between groups 30 and 60 W.

The major morphological findings were the loss of architecture, loss of cellular profiles, and nuclear details (78% of sections). Minor findings related to damage were loss of organ profile, proteinaceous degeneration, and presence of amorphous cytoplasm (34%) of sections. All findings were observed in 45% of cases.

Using the digital microscope vs. optic microscope, almost perfect (*h* index = 0.86) concordance was observed. The assessment of the digital slides (Grundium Ocus) was able to rebuild the 3D images of the damage. The virtual sum up of the ex-glass slide sections resulted in an overall mean depth of damage of 1.1 mm.

The Wilcoxon rank test, *p*-value between groups 30 and 60 W revealed, respectively, a *p-*value of 0.4149 in between 30 vs. 60 W in the group 2 s, and of 0.8345 in between 30 vs. 60 W in the group 4 s.

**Figure 6:**
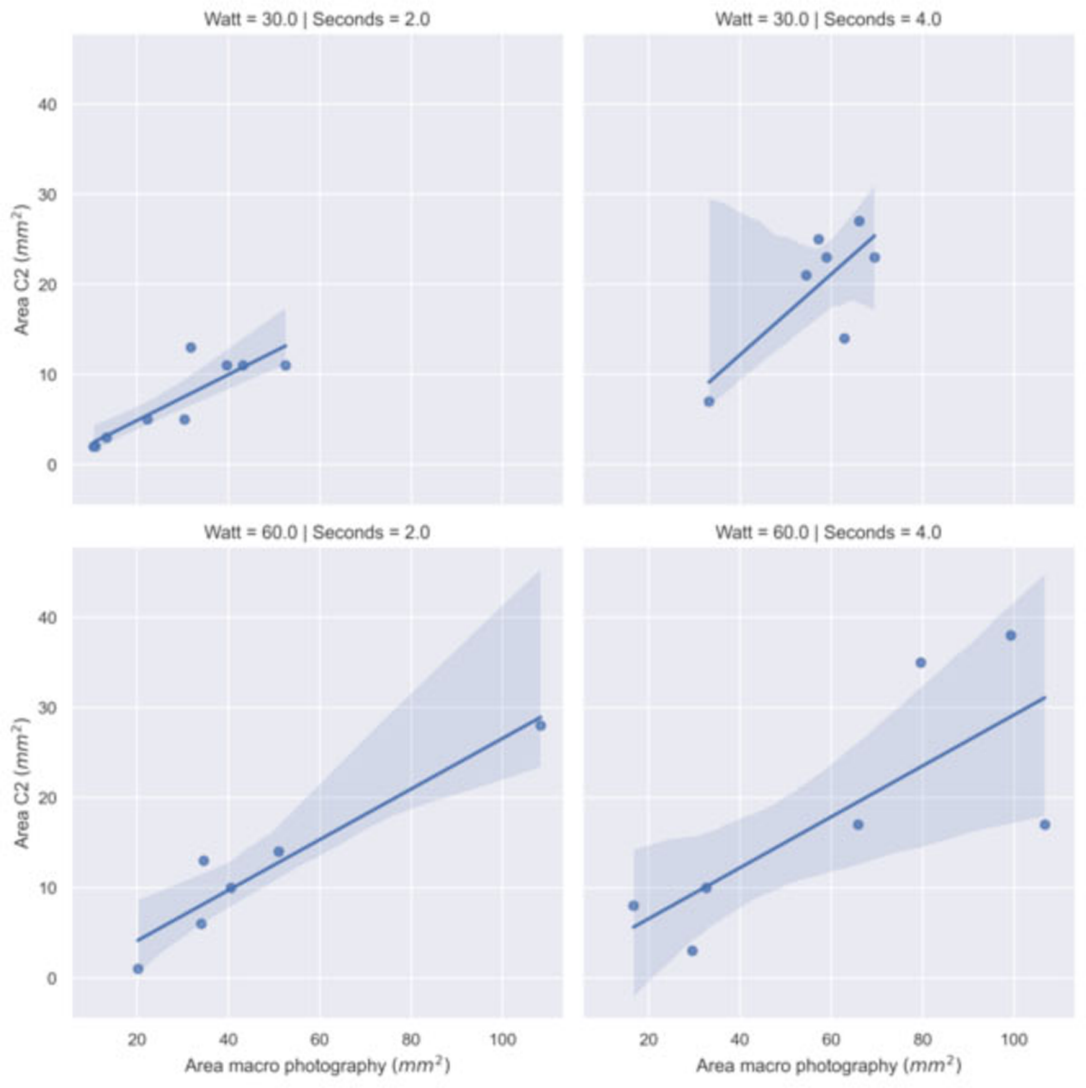
Regression model fit between the area with temperature greater than 60°C recorded by C2 thermal camera and the damaged area recorded by macro photography by power (Watt) and time (second) settings.

We have performed a correlation analysis (Spearman coefficient and regression fit) between the thermal spread estimated with the thermal camera and the size of the surface damage measured by macrophotography. Table 3 and Figure 6 show the results on a subset of samples. The high correlation confirms the consistency with the thermal measurements performed with the C2 camera.

**Table 3:** Spearman correlation coefficients (*r*) between the thermal spread (C2 thermal camera) and the macrophotography of damaged area computed in groups of samples (*N*.) by power setting (watt).

	Area macro photography (mm^2^)			
	*N*	Time: 2 s	*N*	Time: 4 s
Thermal spread (mm^2^)				
Power: 30 W	8	*r* = 0.87208	7	*r* = 0.52254
Power: 60 W	6	*r* = 0.94286	8	*r* = 0.81084

## Discussion

It is well known that monopolar, bipolar, and harmonic energy causes thermal injury to nearby neural tissue and it is associated with a decreased erectile response to a cavernous–nerve stimulation (16). In this way, continuous hemostasis is crucial for laparoscopic and robotic surgeons to maintain good visibility of the surgical field. Alternatively, is possible to use traditional Hem-o-lok clips, but they require the use of additional instruments with an increase in overall surgical times to which one must also add the probable mechanical damage to adjacent sensory and nerve structures.

In the present study, we aimed to evaluate experimentally the thermal energy spread of bipolar cauterizing by a sealed plexiglass chamber reproducing the conditions of a pneumoperitoneum in CO_2_-rich environment at constant pressure and temperature thanks to the use of the compact C2 thermal camera.

It is important to underline that we adopted a compact camera characterized by a small sensor array (therefore, a limited spatial resolution) to test a reliable approach for its deployment in a real environment. Nowadays, miniaturized sensors are available thus addressing the possibility of performing thermal monitoring in robotic-assisted laparoscopic conditions (14). In this study, we showed that thermal sampling with a small 80 × 60 bolometric sensor at an acquisition rate of 10 Hz is suitable to provide informative data for thermal damage monitoring in real time during electrosurgery. This clearly underlines the potential for further studies.

On the technological side, it is important to perform an accurate thermal calibration for assessing the correct threshold to use for identifying the damaged area and the areas at risk. The usage of radiometric sensors with low instrumental drift is hence recommended. Otherwise, a considerable offset between the damaged and monitored area might occur and the thermal images may serve only as a qualitative visual clue that must be interpreted by the surgeon.

This is the first report that aimed to measure the thermal spread of bipolar cauterizing under the conditions of an experimental pneumoperitoneum and allowed us to confirm that even under these particular environmental conditions, the area of heat distribution is closely related to the power of the energy used (Watts) and the energy application time per 2 s.

The application time and the power of the scalpel play a major role in the area affected by the thermal stress. However, other factors might have an impact on the extent of the area affected by necrotization. This fact is highlighted by the presence of outliers and non-normal distribution of the damaged area measured. Further studies are necessary to precisely identify them but from the experience maturated during the experiments, we observed that they may include: tissue's composition variations (for instance, areas with high lipids content), pressure and orientation of the scalpel, presence of tissue residues after consecutive applications of the scalpel on the tissue, as well as the contact surface and opening of the scalpel prongs and, consequently, the amount of tissue between the prongs. Hence, it is difficult for a surgeon to predict the impact of the electrosurgery based on the electrosurgery instrumental parameters used during the operation. For this reason, real-time monitoring of the thermal stress induced by electrosurgery appears to be a valuable contribution during the electrosurgery operation. The data collected highlights a strong correlation between the necrotized area assessed through macrophotography and the area exceeding a threshold temperature of 60°C. There is also a good correlation between the necrotized area assessed through macrophotography and the maximum temperature recorded by the thermal camera. Further studies should be carried out for avoiding biases in the temperature reading that seems to occur with long scalpel application time. Once the technical issues have been solved, the estimation of a cumulative thermal dose will result in a better prediction of the thermal damage. An accurate thermal model and real-time monitoring of electrosurgery collateral effects will then improve the effectiveness of the proposed technique.

Taking into consideration the application at 30 W, the heat distribution area evaluated with the C2 is significantly greater at 4 s than at 2 s. Conversely, this correlation does not appear to be respected when analyzing the same parameters using 60 W. Under the latter conditions (60 W), the absence of a direct correlation between the heat distribution surface and the increase in the released power could be related to the fact that the surface of the tissue damage produced represents a temporary impediment to its further expansion due to tissue and protein denaturation that changes the thermal response. In this context, a further consideration is necessary for thermal depth damage to both the power output and the duration of its release. In fact, it appears that tissue damage would predominantly spread on the surface rather than in depth. The key to understanding this phenomenon is unclear and has never been reported before in the literature by those who have dealt with the subject although the previous contributions were exclusively the result of experimental evaluations applicable to open surgery.

Finally, if we compare our results with those obtained by other authors, albeit under substantially different environmental conditions, we can say that the correlation between thermal spread and energy used is confirmed, as is the energy application time, but with a heat spread at a tissue level that would appear to be more contained within our SPC, probably due to the effect of the constant pressure of CO_2_, although further studies are needed to confirm this hypothesis. Nonetheless, it is worth remarking that our study has some limitations, the first of which is that of not having evaluated the enzymatic assessment of temperature spread, and the second is of not having used a second clamp as a heat sink, even though in current surgical practice this latter solution is difficult to implement in routine mode. As first part of the research project, the experiment was performed on inanimate pig tissue samples using the laparoscopic simulator. Next developments will include experiments on real pigs, thus allowing us to consider the role of physiological processes (such as blood flow) in the heat diffusion throughout the body volumes and in the protection of the tissues from direct damage induced from thermal energy.

In conclusion, we believe that the application of these results is very attractive both for the definition of the careful use of bipolar cautery during nerve-sparing RARP and for the development of a new thermal endoscope for robotic use as designed, patented, and recently published by us (14). In this way, it becomes clear that careful use of bipolar cautery is feasible during posterolateral dissection in RARP procedures allowing the use of the Hem-o-lok clip whenever possible.

## Data Availability

The raw data supporting the conclusions of this article will be made available by the authors, without undue reservation.
